# Jacek Namieśnik—Analytical Chemist and Dedicated Biker: From Wine Analysis to Toxic Compounds

**DOI:** 10.3390/molecules26123536

**Published:** 2021-06-09

**Authors:** Jerzy Silberring, Pawel Ciborowski

**Affiliations:** 1 Department of Biochemistry and Neurobiology, AGH University of Science and Technology, 30-059 Krakow, Poland; 2 Department of Pharmacology and Experimental Neuroscience, University of Nebraska Medical Center, Omaha, NE 985800, USA

Jacek Namieśnik, who died at the age of 69, was one of the most influential analytical chemists in Poland at the second half of the 20th century and the first two decades of the 21st century. He was dedicated to analysis using a broad range of methods. His scientific collaborations include scientists from not only from Poland but also from many countries worldwide. His achievements, both scientific and organizational, resulted in over 359 publications (PubMed), and this does not include the books he edited, and book chapters he contributed to. It is by all means impressive. He was also a dedicated teacher and mentor of many doctorate theses. The hallmark of his work was environmental and food analyses. Here, we would like to focus on his research, which has been partially related to our work and devoted to human health. Detailed analysis of many toxic compounds was performed by his group, including metals and biogenic amines in wine samples [1,2].

In addition, body fluids, taken in a non-invasive manner, were investigated [3]. This resembles, to some extent, our work on identification of body fluids, such as proteinergic profiles, novel neuropeptides, enzymes, and their inhibitors [4].

Another important contribution of Jacek Namieśnik to analytical chemistry was the validation of analytical techniques and the foundation of the first Polish dictionary of analytical abbreviations and terms.

Prof. Namieśnik was a connoisseur of liquors, and as a thoroughbred analyst, he felt an obligation to check the content of bottles before consumption. This passion resulted in several publications describing analytical techniques used in the examination of wines and whiskeys. This chapter of his scientific interest seems to be thorough and detailed, also including home wine production, as in the papers [5,6]. All those papers involve various types of mass spectrometry, among other methodologies. International authorship Polish–Bulgarian–Greek–Spanish, ensures proper quality of the products tested. Preferentially, biogenic amines were in focus. Luckily, biogenic amines are not poisonous, except from a higher content of histamine, mostly in the spoiled fish. Biogenic amines are products of the decarboxylation of amino acids by yeasts during fermentation, but other pathways can also be a contributing factor. Storage of the wines was also of special interest [2] and both white and red sorts that were kept at ambient and +4 °C up to 30 days. The most important conclusion was that histamine level decreased with time, but our recommendation coming from our own, long-term experience would be to drink wine right to the bottom directly after opening. Some striking fact is that neither Bulgarians nor Greeks did not contribute with their really good bottled products. Two publications deal with the metals content in the homemade wines. The results are reassuring that our own legal products can be drunk safely [1,2,5].

A set of papers describes detailed analysis of whisky [6,7,8]. In one of the publications the authors conclude that the biggest differences between different types of whisky are due to their aroma [7]. We agree with this observation to some extent, but Prof. Namieśnik, as a connoisseur, certainly was aware also of the taste. Electronic nose, i.e., the ultrafast GC/MS technique proved very useful for differentiation of various strong alcohols (vodka and whisky), and the usefulness of this device was tested on a large number of samples [8]. The good news is that such a simple technique is capable of differentiation between alcohols but also allows for the detection of eventual forgeries. It is a pity that moonshine samples were out of the scope of this project. Differentiation between various origins of whisky was published in [9]. Several sorts of spirits were tested by means of the headspace mass-spectrometry, mid infrared, and UV-vis spectroscopy. Again, it was possible to differentiate between Scotch, Bourbon, Tennessee, and Spanish (!!), however we will stay struggling as one of us prefers bourbon and the other Scotch.

**Jacek Namieśnik and mass spectrometry.** In his work, Prof. Namieśnik equally focused on every step of analysis, which usually included sample preparation, separation such as liquid chromatography, and detection steps. He used his talent as an analytical chemist to optimize many methods providing others with new tools to investigate important samples such as food or environment [10,11], just to mention few of such reports from his early work. Based on PubMed listing, he published 85 peer-reviewed papers, which included mass spectrometry. It is important to note that he always chose detection methods that were the most suitable for the task. One such example was the use of GC-MS. His work in this area can be divided into two avenues. One is development and/or improvement of analysis such as [12,13]. In recent years, he also contributed to analysis of components of e-cigarettes, as it was emerging as a health issue worldwide [14,15,16]. These three citations showed him as an emerging scientist to apply his analytical expertise to new challenges as manufacturers moved from combustible tobacco to other forms leading to addiction. His vision as an analytical chemist was focused on the safety of flavoring additives added to e-cigarettes when he published his paper in the Journal of Chromatography A in 2017 [16]), ahead of reports of lung complications resulting from vaping and a ban on certain flavors issued in some part of the United States. As a scientist, he focused in this publication on 88 additives, but more importantly, he put emphasis on validation. This is by far the most comprehensive study in this area.

His master approach to analytics with an excellent combination of sample preparation and chromatographic conditions, allowing the detection of so many analytes in one analytical run [15]. We can only hope that his work will be continued by one of his students that he trained.

**Jacek Namieśnik as visionary educator**. Diversity in educational experiences was a driving force in his vision of enhancing education. Jacek saw limitations in sending all graduate students abroad to experience various styles of and topics in education. He did not wait and actively invited established scientists to come to the Department of Chemistry of Gdansk University of Technology. Invited lecturers were mandated to provide specific courses for graduate students in a short period of time and lecture in English. Being one of them between 2011 and 2019 and lecturing to students from the Gdansk area, I saw an extraordinary benefit and opportunity of graduate students to enhance their experience in direct contact with the international scientific community. Big achievement, no doubt.
